# A comprehensive characterisation of natural aerosol sources in the high Arctic during the onset of sea ice melt†

**DOI:** 10.1039/d4fd00162a

**Published:** 2024-11-25

**Authors:** Gabriel Pereira Freitas, Julia Kojoj, Camille Mavis, Jessie Creamean, Fredrik Mattsson, Lovisa Nilsson, Jennie Spicker Schmidt, Kouji Adachi, Tina Šantl-Temkiv, Erik Ahlberg, Claudia Mohr, Ilona Riipinen, Paul Zieger

**Affiliations:** a Department of Environmental Science, Stockholm University Stockholm Sweden paul.zieger@aces.su.se; b Bolin Centre for Climate Research, Stockholm University Stockholm Sweden; c Department of Atmospheric Science, Colorado State University USA; d Department of Physics, Lund University Sweden; e Department of Biology, Aarhus University Denmark; f Department of Atmosphere, Ocean, and Earth System Modeling Research, Meteorological Research Institute Tsukuba Japan; g PSI Center for Energy and Environmental Sciences, Paul Scherrer Institute Villigen Switzerland; h Department of Environmental Systems Science, ETH Zurich Zürich Switzerland

## Abstract

The interactions between aerosols and clouds are still one of the largest sources of uncertainty in quantifying anthropogenic radiative forcing. To reduce this uncertainty, we must first determine the baseline natural aerosol loading for different environments. In the pristine and hardly accessible polar regions, the exact nature of local aerosol sources remains poorly understood. It is unclear how oceans, including sea ice, control the aerosol budget, influence cloud formation, and determine the cloud phase. One critical question relates to the abundance and characteristics of biological aerosol particles that are important for the formation and microphysical properties of Arctic mixed-phase clouds. Within this work, we conducted a comprehensive analysis of various potential local sources of natural aerosols in the high Arctic over the pack ice during the ARTofMELT expedition in May–June 2023. Samples of snow, sea ice, seawater, and the sea surface microlayer (SML) were analysed for their microphysical, chemical, and fluorescent properties immediately after collection. Accompanied analyses of ice nucleating properties and biological cell quantification were performed at a later stage. We found that increased biological activity in seawater and the SML during the late Arctic spring led to higher emissions of fluorescent primary biological aerosol particles (fPBAPs) and other highly fluorescent particles (OHFPs, here organic-coated sea salt particles). Surprisingly, the concentrations of ice nucleating particles (INPs) in the corresponding liquid samples did not follow this trend. Gradients in OHFPs, fPBAPs, and black carbon indicated an anthropogenic pollution signal in surface samples especially in snow but also in the top layer of the sea ice core and SML samples. Salinity did not affect the aerosolisation of fPBAPs or sample ice nucleating activity. Compared to seawater, INP and fPBAP concentrations were enriched in sea ice samples. All samples showed distinct differences in their biological, chemical, and physical properties, which can be used in future work for an improved source apportionment of natural Arctic aerosol to reduce uncertainties associated with their representation in models and impacts on Arctic mixed-phase clouds.

## Abstract 2

A comprehensive analysis of various potential local sources of natural aerosols in the high Arctic over the pack ice during the ARTofMELT expedition in May–June 2023 was conducted.

## Graphical abstract

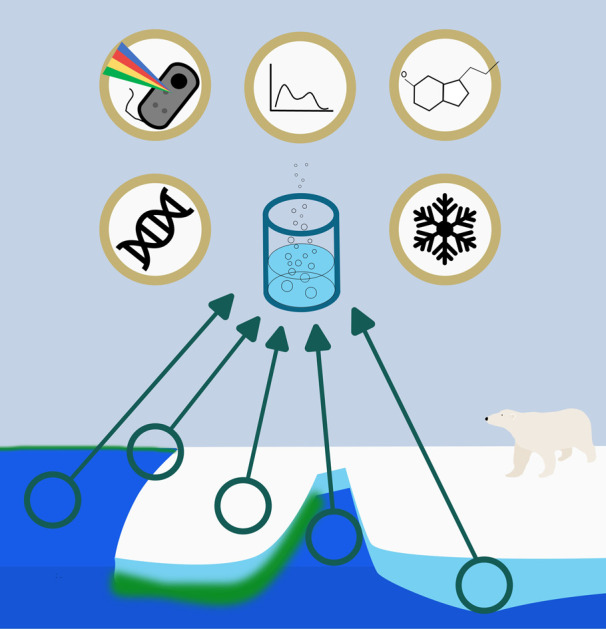

## 1 Introduction

Climate change is manifested most in the rapidly changing Arctic. Here, the observed temperature increase is almost four times higher than the global average with local amplification of up to six or even seven times.^1^ This rapid warming, known as Arctic amplification,^2^ is interlinked with the Earth's climate system and has consequences on global weather systems^3^ and the climate within and outside the Arctic.^4^

**Fig. 1:**
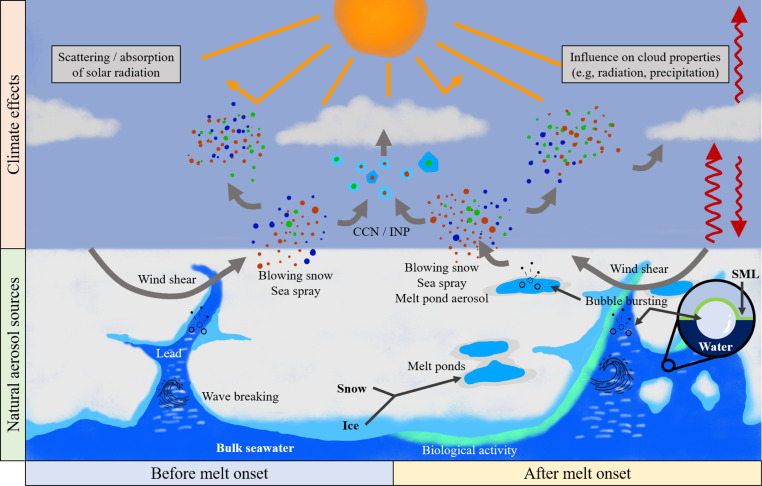
Sources of natural primary aerosol particles over the high Arctic Ocean during the onset of sea ice melt. Primary particles can be lofted to the atmosphere *via* wind stress on surfaces (*e.g.*, blowing snow, wave breaking) and bubble bursting (*e.g.*, in leads or melt ponds). Once in the atmosphere, particles can interact with solar radiation and influence cloud properties by acting as cloud condensation nuclei (CCN) or ice nucleating particles (INP).

Aerosols, suspended liquid or solid particles in the air, are known to influence the Arctic climate by affecting solar radiation, cloud formation, and atmospheric composition (see Fig. 1). These particles can either be directly emitted into the atmosphere (primary aerosols) or formed from gaseous precursors (secondary aerosols). In winter and spring, the Arctic aerosol population is dominated by long-range transport of particles from lower latitudes, including biomass burning^5^ or pollution aerosols from anthropogenic sources.^6^ This phenomenon is also known as Arctic haze.^7,8^ In late spring, the shift in atmospheric dynamics induced by the increase in solar radiation blocks northward aerosol transport to a large degree, and local aerosol sources instead drive changes in the aerosol population.^9–12^ Summertime sources include secondary aerosols,^13–15^ such as the oxidation of dimethyl sulphide, common in marine environments. In addition, aerosols from the free troposphere and entrainment from above the stable boundary layer could contribute to aerosol particles in the high Arctic during summer.^16^ Primary aerosols are also significant in the Arctic, defined here as the region above the Arctic Ocean's pack-ice, including the biologically active marginal ice zone (MIZ). Key sources during the summer are sea spray from breaking waves in open water^17,18^ and terrestrial emissions from the northern continental regions or islands in the Arctic.^19–21^ Blowing snow is another significant polar primary aerosol source,^22,23^ but it is more prominent during winter.^24^ A crucial category of primary aerosols are those of biological origin – spores, bacteria, pollen, viruses, or algae – found in the ocean, sea ice, or snow. These are known as primary biological aerosol particles (PBAPs) and are vital to the Earth's biosphere, climate system, and hydrological cycle.^25–28^

Clouds significantly affect the surface energy budget in the Arctic,^29,30^ influencing the melting and freezing of sea ice.^31–34^ Unlike mid-latitude or subtropical clouds, which generally cool the climate, with the exception of the middle of summer, low-level Arctic mixed-phase clouds (AMPCs) warm the surface^35,36^ and can persist throughout the year for days or weeks at a time.^37^ The impacts of AMPC radiative properties on sea ice albedo is further influenced by aerosol particles, both natural and anthropogenic, which act as cloud condensation nuclei (CCN) or ice nucleating particles (INPs).^38–40^ The inherently complex AMPCs have proven particularly difficult to accurately reproduce in models,^37,41,42^ and the relevant sources of natural aerosols, and understanding their interactions with clouds, remain incomplete pieces of the puzzle.^38,43,44^ In fact, on a global scale, natural aerosol sources and properties are the largest source of uncertainty in estimates of the cloud radiative forcing induced by aerosols.^45^

In summer, high Arctic low-level clouds are often optically thin due to fewer, larger droplets or ice crystals.^46^ This is linked to the pristine air with very low CCN concentrations, as anthropogenic influence is limited.^47,48^ Consequently, marine particles from the marginal ice zone and further north serve as CCN.^13,49,50^ INP observations in the Arctic are more limited than CCN, yet recent studies have targeted evaluation of their quantities and sources, including over the full sea ice annual cycle.^51^ Despite the known importance of CCN and INPs for cloud processes, and the body of recent work focusing on CCN and INPs, their abundance, sources, atmospheric transformations, and sinks in the Arctic remain insufficiently understood,^15,52,53^ yet are critical for climate prediction.^54^

The interface between the ocean and atmosphere, known as the sea surface microlayer (SML), is enriched in biogenic organic material, including different and distinct microbial communities,^55,56^ which can be released through bubble bursting *via* sea spray.^57–59^ Sea spray is composed of inorganic sea salt particles, which can be coated by water-dissolved organics and/or accompanied by primary biological particles.^60^ The biological and organic composition of sea spray aerosol is thus linked to the ocean's physico-chemical and biological state, with biological particles such as marine gels potentially acting as a significant source of CCN.^61,62^ In a marine and Arctic environment, PBAPs may serve as a significant source of INPs,^21,51,63–66^ thus playing a significant role in modulating cloud radiative properties and precipitation formation.^67–69^ Snowfall effectively removes PBAPs from the atmosphere,^70^ but some microorganisms may survive and potentially grow within the snow-pack,^71^ and possibly be re-suspended through blowing snow^23,72^ although this has yet to be confirmed by observations. Microorganisms are also trapped in sea ice,^73^ where they can thrive, and are released into seawater or melt ponds when the ice melts,^74^ where they are possibly emitted as aerosols through bubble bursting or wave breaking.^75^ Non-wind driven mechanisms such as algae respiration bubbles^76^ and bubble inclusion releases in the ice^77^ were discussed by Beck *et al.* in 2024,^72^ but the extent of their contribution is hard to quantify.

Recent studies suggest PBAPs are a dominant source of high-temperature INPs (*i.e.*, INPs that can form cloud ice at temperatures ≥−15 °C) in the Arctic,^21,72^ contributing to uncertainties in INP predictions and cloud properties in models.^78^ Creamean *et al.*^79^ found a seasonal increase in airborne biological INPs during the high Arctic summer, coinciding with high melt pond and lead fractions in the sea ice, pointing to a biological INP source in the marginal ice zone (MIZ). Further support comes from fluorescent particle observations, which also peaked during this period, indicating a local marine source.^72^ Close to Arctic land masses, the vegetation and soil seem to be the main sources for PBAPs and INPs,^21,80^ but it is unclear how much they contribute to their high Arctic respective populations. Thus, one way to assess the ratio between local and transported sources is first characterising the local aerosol sources.

To incorporate biological INPs into models, sources and emission processes must be clarified.^68,78^ Open-water features within sea ice, such as leads and melt ponds, are potential sources, with biological activity influenced by the age and thickness of sea ice and snow,^81,82^ and the formation of leads.^83^ However, differentiating between various sources of PBAPs and INPs – sea ice, seawater, or snow – requires more specific characterisation.^72^

The purpose of this study is to analyse key potential natural sources of aerosols in the high Arctic, focusing on sea ice, snow, and seawater, during the transition from spring to summer (*i.e.*, up to the melt onset). As sea ice retreats and as the melt season is lengthened, new natural aerosol sources will emerge, making it crucial to understand their origins and properties for more accurate climate predictions in the Arctic. By studying the biological, physical, and chemical characteristics of these aerosols, we aim to gain deeper insights into their role in Arctic climate processes, particularly in poorly understood areas such as aerosol–cloud interactions.

## 2 Methods

### 2.1 The ARTofMELT expedition

The ARTofMELT (atmospheric rivers and the onset of sea ice melt) expedition on board the Swedish icebreaker I/B *Oden* took place from May 7th to June 15th 2023. One main scientific goal of the expedition was to study the various atmospheric, sea ice and ocean processes that are important during the onset of the sea ice melt season, including, among others, the role of aerosols and clouds. “Source” samples were taken throughout the expedition at two dedicated ice floe camps, where *Oden* was moored to an ice floe, and predominantly from remote ice stations accessed by helicopter. The sites were chosen in order to obtain samples from different areas with a range of sea ice thickness, open water sources (leads), and snow coverage (see Fig. S1 and Table S1 in the ESI†).

### 2.2 Source sampling

Samples of seawater from leads, sea ice cores, snow, and the sea surface microlayer (SML) were retrieved simultaneously at various sampling sites throughout the expedition. Sea ice cores (7.25 cm in diameter) were collected using a Kovacs Mark III Ice Coring System, permeating the measured thickness of the sea ice at the sampling location (see Fig. S1†). Segments of 10–20 cm were cut from the top, middle, and bottom of the ice cores. Each segment was placed in a separate Whirl-Pak® bag.

Snow samples were collected by digging snow pits near the ice coring locations from the top of the snowpack to the sea ice surface (see Table S2† for depths). Temperature profiles were measured every 10–20 cm. Snow was collected in separate containers for each analytical method directly from the top 10 cm and bottom 10 cm of the snow pit (if deep enough).

Lead water samples were collected in individual containers at the surface and at 5 or 10 m depth using a horizontal water sampler (Pentair), then poured into containers that were triple-rinsed with lead water. When present, “slush” in the open leads, or porous sea ice broken off from the floe edge and/or refrozen surface ocean water, was also skimmed from the surface and collected into containers. Water samples were stored frozen on *Oden* until analysis.

SML samples were collected by submerging a 28 × 52 cm glass plate vertically through the SML and slowly retracting it (5 cm s^−1^), allowing the SML to adhere to the glass surface.^84^ The adhered SML was then scraped off into individual containers. The glass plate was cleaned before and after sampling by rinsing with 70% ethanol and ultra-pure water (MilliQ, Direct Q3, Merck).

Ice cores remained frozen on *Oden* until analysis, when they were melted at room temperature then partitioned in separate containers for the various analyses described below. All other samples that were collected in their separate containers at each ice station were stored frozen on board *Oden* and successively analysed within 1–2 days using the aerosol *in situ* instruments installed in the Stockholm University Department of Environmental Science (ACES) mobile laboratory. Duplicates of the same samples were stored and transported frozen for biological characterisation and ice nucleating particle analysis after the expedition ended at Aarhus University and Colorado State University, respectively.

### 2.3 Source sample analyses

#### 2.3.1 Aerosol generation for analyses on-board *Oden*

On-board analyses of melted samples took place 1–2 days after collection, whereby aerosols were generated *via* atomisation, and measurements were taken using a multiparameter bioaerosol spectrometer (MBS), scanning electrical mobility spectrometer (SEMS), soot particle aerosol mass spectrometer (SP-AMS), single particle soot photometer (SP2), and a transmission electron microscopy analysis (TEM) grid sampler.

**Fig. 2:**
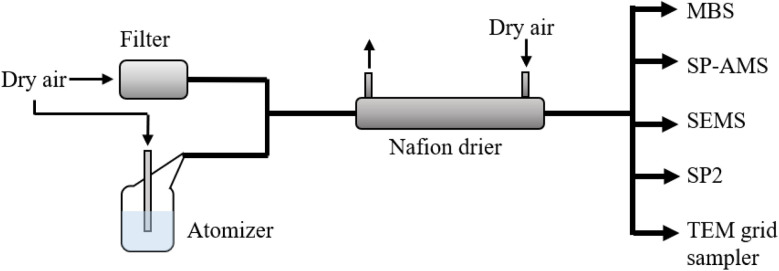
Set-up of the experiment. Aerosol particles were atomised using an atomiser, the aerosols were diluted with dry particle-free air and successively analysed using the multiparameter bioaerosol spectrometer (MBS), soot-particle aerosol mass spectrometer (SP-AMS), single-particle soot photometer (SP2) and scanning electrical mobility spectrometer (SEMS), and using filter sampling with transmission electron microscopy (TEM) analyses performed after the expedition. The relative humidity was monitored at the inlet of the SEMS.

An aerosol generator (Model ATM 228, Topas GmbH, Germany) was used to atomise particles from the melted samples. The particle stream was diluted with particle-free air and dried using a Nafion dryer (Model MD-700, Perma Pure, USA) to a relative humidity of RH = 22 ± 3.2% before being split into the different aerosol samplers (see Fig. 2). The sampling bottles of the aerosol generator were thoroughly cleaned before and after each experiment using isopropanol and ultra-pure water (MilliQ, Direct Q3, Merck) and rinsed several times with ultra-pure water. Leak and background tests were performed regularly by injecting particle-free air and running the atomiser with an empty rinsed bottle.

The atomiser operates by creating a differential pressure between the air in the sample bottle and the incoming particle-free air that enters through the nozzle of the aerosol generator, which is submerged in the liquid sample. This creates a negative pressure at a sample inlet in the nozzle, causing the sample to flow into it and converge into droplets that exit the nozzle and rise to the surface of the sample inside bubbles. The aerosolised sample is released through bursting bubbles at the sample surface, thus to some extent mimicking natural sea spray.^85^ The particle concentration produced depends on the differential nozzle pressure set, which determines the air flow.

In this work, we were primarily interested in characterising fluorescent primary biological aerosol particles (fPBAPs) in the coarse mode, as they are most likely to be relevant as INPs, and because this is the size range where they can be detected online with the MBS. Therefore, the pressure setting of the atomiser was regulated to generate rates of particles above >0.8 μm in diameter that were sufficient for detection, while keeping the pressure as low as possible to achieve as gentle and realistic atomisation as possible. Due to the large variation in salinity between source types, the pressure had to be adjusted for each sample. Samples with higher salinity like seawater from the leads reached appropriate particle rates at lower pressures, while it had to be increased for samples with lower salinity like snow (see Fig. S2†). For samples of the same source type (*e.g.*, all snow samples), the nozzle pressure was constant throughout the entire set of experiments. To ensure that aerosolising the samples at different pressures did not have unexpected effects on the generated particle concentration in the fine or coarse mode, respectively, we ran two experiments with the sample types with lowest salinity, sea ice and snow, with the atomiser pressure increased step-wise to cover the entire range of settings used during all experiments (see Fig. S2†). The air flow from the atomiser to the instrumental setup was kept constant by the pumps behind the instruments, and an added make-up flow of dry particle-free air (see Fig. 2). The total sample flow rate between the atomiser and all sampling instruments was 3.7 L min^−1^.

#### 2.3.2 The multiparameter bioaerosol spectrometer (MBS)

An MBS (University of Hertfordshire, UK) was used to determine the size, shape and fluorescence characteristics of particles above *D* > 0.8 μm (optical diameter) on a single-particle basis. A UV-flashlight was used to excite fluorescence at 280 nm wavelength and the emitted fluorescence was measured at eight different channels between 305 and 655 nm using a spectrometer. Using a low- and a high-power infrared laser, the MBS also determines the optical diameter and 2D-scattering pattern. The latter holds information on the morphology of the detected particle. More technical details can be found in Ruske *et al.*^86^

The MBS was specifically designed to detect biological particles using fluorescence. The classification of fluorescent particles follows a decision tree described by Freitas *et al.*^59^ in 2022. In summary, each fluorescence emission channel is treated individually and assigned two thresholds. The first threshold (3 times the background signal, measured every 30 000 particles and before the actual measurement started) classifies particles as fluorescent particles (FP), while the second (9 times the background) classifies them as highly fluorescent particles. These particles are assigned a spectral class based on the channels where their signal exceeds the second threshold, represented by letter combinations (A–H, for each channel). Particles with the highest signal in channel B (364 nm) are classified as fluorescent primary biological aerosol particles (fPBAPs), while others are grouped as other highly fluorescent particles (OHFPs). The MBS's channel B specifically detects tryptophan fluorescence, a marker for microorganisms. fPBAP spectral classes B, BC, ABC, and ABCD are referred to as fPBAP types I–IV, with other classes grouped as type V. The average fluorescence spectra for fPBAPs and the most abundant OHFPs are shown in Fig. S3† (see also Freitas *et al.*^21^).

#### 2.3.3 The scanning electrical mobility spectrometer (SEMS)

The sub-micron particle number size distributions were measured using a SEMS (Model 2100, Brechtel Inc. USA). The particle stream passed through an impactor to remove particles above 1 μm (round jet impactor, model 8009, Brechtel Inc. USA) before being charged using a Ni-63 bi-polar charger. A differential mobility analyser (DMA) and a mixing condensation particle counter (MCPC, model 1720, Brechtel Inc. USA) were then used to scan the size distributions between 0.005 and 1 μm (electrical mobility diameter) every 1 min. The multiple charge and loss correction within the SEMS was performed with the provided manufacturer's software. A second MCPC (Model 1720, Brechtel Inc. USA) was used in parallel to measure the total concentration of sub-micrometre particles. The total sampling flow rate of the SEMS and MCPC was 0.76 L min^−1^. The sizing of the SEMS was verified using polystyrene latex spheres of known sizes (100 nm and 269 nm, respectively).

#### 2.3.4 The soot particle aerosol mass spectrometer (SP-AMS)

A SP-AMS (Aerodyne Inc., USA) was deployed to determine the chemical composition of sub-micron particles. The SP-AMS was alternating between a laser on and a laser off mode. During laser on, an intra-cavity Nd:YAG laser (1064 nm) was used to vaporise the soot particles. In laser off mode, only the standard tungsten vaporiser, set to 600 °C, was used to measure the non-refractory composition. The aerosol components were ionised, using 70 eV electron ionisation, and subsequently entered the mass spectrometer in order to detect their chemical composition in real-time. More technical details can be found in Onasch *et al.*^87^ Within this work, only the non-size-resolved laser off data was used. It includes the mass concentration of non-refractory material such as organics, nitrate, chloride, ammonium and sulphate. All SP-AMS data were processed and analysed with software packages SQUIRREL 1.66 and PIKA 1.26. Mono-disperse 300 nm (using the DMA from the SEMS) ammonium nitrate particles were used for the calibration of the ionisation efficiency.

#### 2.3.5 The single particle soot photometer (SP2)

A SP2 (Droplet Measurement Technology, Boulder, USA) was used to determine the refractory black carbon (rBC) mass of single particles. More technical details can be found in Schwarz *et al.*^88^ and Stephens *et al.*^89^ In the SP2, single particles cross a continuous wave intracavity laser (Nd:YAG,1064 nm) carried by a sample flow (0.12 L min^−1^) and constrained by a sheath flow. Absorbing particles, such as soot, are brought to incandescence, which yields a signal related to the single-particle mass. Here, the SP2 incandescence detectors were calibrated with Aquadag® and recalculated to a fullerene soot equivalent following Laborde *et al.*^90^ The SP2 can determine the rBC mass between 0.3–117 fg corresponding to a volume equivalent diameter of 70–500 nm (assuming a density of 1.8 g cm^−3^). However, below 0.9 fg, the detection efficiency is reduced below 100%.^90^

#### 2.3.6 Transmission electron microscopy (TEM) analysis

Coarse- and fine-mode particles with aerodynamic diameters larger and smaller than 0.7 μm, respectively, were sampled on transmission electron microscopy (TEM) grids using an impactor sampler (AS-24W, Arios Inc., Tokyo, Japan). The morphology and composition of individual particles from selected coarse-mode TEM samples (one sample of sea ice and lead water and two samples of snow) and the morphology of all fine-mode samples were analysed using a transmission electron microscope (JEM-1400, JEOL, Tokyo, Japan) equipped with an energy dispersive X-ray spectrometer (EDS; X-Max 80, Oxford Instruments, Tokyo, Japan). The technical details of the TEM sampling and analysis are described in Adachi *et al.*^91^

#### 2.3.7 DNA extraction and quantitative polymerase chain reaction

DNA was extracted from a selection of source samples following the DNeasy® PowerSoil® Pro Kit protocol (HB-2495-002 ©2018, Qiagen). Two modifications were applied to the protocol. In step 2, the PowerBead Pro Tube was vortexed in a TissueLyzer (Qiagen) for 10 min at a speed of 40 Hz. In step 16, 50 μL of solution C6 was added to the filter membrane, followed by 10 min of incubation to ensure a higher DNA yield from the extraction. To quantify the amount of bacterial 16S rRNA and eukaryotic 18S rRNA gene copies, a quantitative Polymerase Chain Reaction (qPCR) was performed on a 2 μL DNA template. Samples were run on a MX3005p qPCR instrument (Agilent, Santa Clara, CA, United States). To target the 16S rRNA gene sequence, the universal primers Bac908F (5′-AAC TCA AAK GAA TTG ACG GG-3′) and Bac1075R (5′-CAC GAG CTG ACGACA RCC-3′) were used following the methods described by Lever *et al.*^92^ Some modifications were applied to the qPCR protocol, including (1) 95 °C polymerase activation for 15 min, followed by (2) 40 PCR cycles, (3) elongation for 15 s and (4) acquisition for 15 s. The number 18S rRNA gene copies were quantified using primers Euk345F (5′-AAGGAAGGCAGCAGGCG-3′) and Euk499R (5′CACCAGACTTGCCCTCYAAT-3′) following the methods described by Zhu *et al.*^93^

#### 2.3.8 Ice nucleating particle analysis

All samples were processed on the Colorado State University Ice Spectrometer (IS)^94^ 5–14 months after collection. Details of processing source samples are found in Barry *et al.*,^95^ but are described briefly here. The solutions were prepared by thawing the collected samples at room temperature without further processing. The IS contains two 96-well temperature-controlled aluminium blocks fitted with disposable clean PCR trays that enable the analysis of two samples at a time (11-, 121- and 1331-fold, and occasionally in higher dilutions of 20-, 400-, and 8000-fold dilutions plus a 0.01 μL-filtered deionized water blank). For each sample, aliquots of 50 μL were dispensed into the PCR tray wells in a laminar flow clean hood, the trays were placed in the aluminium blocks in the IS, the blocks were covered with a plexiglass window, and the head space purged with cooled, dry, particle-free N_2_. Frozen wells were counted at each 0.5 °C interval as the temperature was lowered at ∼0.33 °C min^−1^ to ∼−30 °C. The cumulative concentrations of INPs per mL of sample were calculated using the equation from Vali^96^ in 1971. In total, 37(15) ice core segments, 23(11) snow, 31(18) lead water, and 8(6) SML samples were analysed (analysed) on the IS (see also Table S1†).

#### 2.3.9 Supporting analyses and data

The *Oden* is equipped with a FerryBox I system (-4H-JENA engineering GmbH, Jena, Germany) for the continuous analysis of key underway seawater properties such as temperature, salinity or turbidity. The water intake is at approximately 8 to 9 m below sea level (depending on the current draught of the ship). Here, we used the chlorophyll-*a* data measured using a WetLabs ECO FLNTU(RT) sensor within the FerryBox I system. Since the absolute calibration of the sensor was lacking, we show the normalised signal in conjunction with chlorophyll-*a* values from discrete filter samples from a fully calibrated fluorometer (see next paragraph).

The source samples were also probed for their chlorophyll-*a* concentrations. Chlorophyll-*a* was measured using a benchtop laboratory fluorometer (Trilogy, Turner Designs, Inc.) with a chlorophyll-*a* non-acidification module. For preparation, at least 500 mL (up to 2000 mL) of each source sample were filtered through borosilicate glass grade GF/F filter discs (Whatman®, 0.7 μm pore size) through a filtration assembly (PYREX 47 mm microfiltration all-glass assembly) under vacuum. Each filter was added to 2.5 mL of 90% acetone and stored in 15 mL centrifuge tubes at −20 °C in the dark for 24 hours. After 24 hours, the suspension was thawed in the dark at room temperature, after which the filters were compressed at the bottom of the tube with an acetone-cleaned spatula. The supernatant was extracted with a 5 mL pipette into a 12 × 75 mm borosilicate test tube (Fisher). The tube was placed into a tube adapter in the Trilogy® and analysed for chlorophyll-*a* concentration (μg L^−1^). After analysis, the glass tubes were triple rinsed with acetone for reuse.

Salinity was measured by first recovering the conductivity of the source samples using a EXTECH ExStikII EC400 TDS/Conductivity/Salinity Pen and using the practical salinity scale (PSS-78) with Hill-86 modification.^97^

## 3 Results and discussion

### 3.1 The increase in biological activity preceding the onset of sea ice melt

**Fig. 3:**
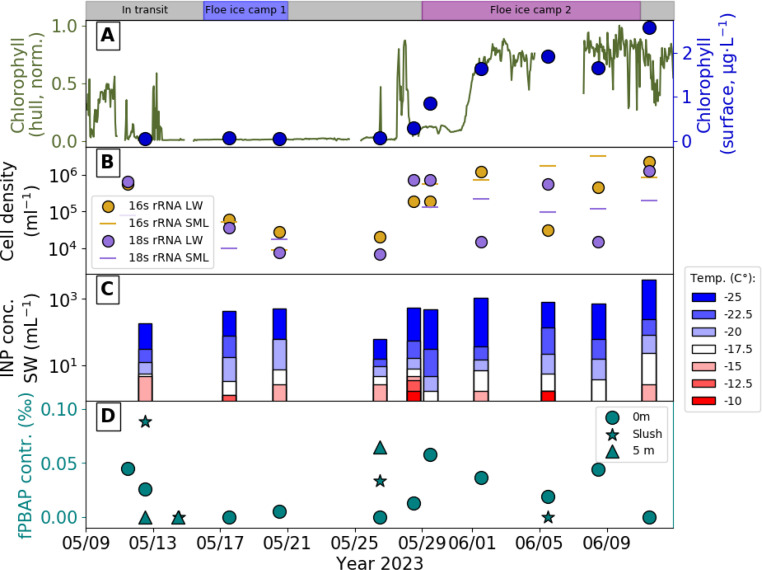
Change in biological activity within the seawater throughout the expedition. Top of (panel A) shows the different sampling periods (in transit and floe ice camps). (A) Continuous normalized chlorophyll-*a* concentration measured by the ship's clean sea water supply (ship hull) and chlorophyll-*a* measured from the surface lead water samples at the remote ice stations. (B) 16S rRNA (prokaryote) and 18S rRNA (eukaryote) gene copies per mL sample of sea surface microlayer (SML) and lead water from the remote ice stations. (C) Ice nucleating particle (INP) concentration as measured from surface lead water for distinct freezing temperatures. (D) Contribution (in ‰) of fluorescent primary biological aerosol particles (fPBAPs) to the coarse mode (particle diameter >0.8 μm) aerosol generated using surface lead water, surface lead slush and lead water collected at a depth of 5 m from leads at the remote ice stations.

A sudden increase in biological activity within seawater was observed at the end of May by a clear increase in chlorophyll-*a* concentration in the continuously measured underway seawater system on *Oden* and in the analysed discrete surface lead water samples collected at the remote ice stations (see Fig. 3A). Chlorophyll-*a* values at the surface reached values of around 2 μg L^−1^, which are higher than those found at the end of the Arctic summer.^79,98^ This elevation preceded the melt onset by two weeks, which happened around the 10th of June 2023. It also coincided with transiting and mooring to the main floe of the second ice camp, which was co-located with much shallower ocean waters. Shallower water and shelf breaks are subject to enhanced marine productivity, due to access to more nutrients from freshwater fluxes (that is, river discharge and glacial melt) and upwelling from warmer Atlantic waters.^99–102^ The spike in biological activity is also reflected in a sudden increase in the cell density of bacteria and algae (see Fig. 3B). In contrast, the INP concentration (see Fig. 3C) in the lead water, at high temperatures of −15 °C or above, did not appear to be significantly affected by this transition. At −25 °C, the INP concentration appears to vary similarly to fPBAP and cell density, but less dramatically. The total concentrations of INPs before and after the increase in biological activity are within the same order of magnitude (see Fig. S4A†), indicating that despite the increase in cell concentrations, microorganisms capable of nucleating ice, especially at higher temperatures, did not increase in population during this period. This is in line with previous studies that have found that INPs increase more during bloom decay; that is, they have a delayed response to the surge in biological production.^51,103,104^ A similar effect has also been observed over land by Freitas *et al.*,^21^ who saw an increase in INP concentration in Svalbard first once there was no snow on the terrestrial surfaces.

The contribution of fPBAPs to the coarse-mode aerosol (particle diameter >0.8 μm) in the atomised lead water samples (see Fig. 3D) increased after the start of biological activity was observed in both the underway seawater and lead water, especially for aerosol generated from surface lead water samples. This observation aligns with the increase in cell concentrations in both surface lead water and SML. Thus, for the period immediately preceding the onset of melt, the increased biological activity was reflected in the aerosolised bioaerosols from the samples. However, this increase may have had a limited effect on INP concentration.

These observations do not imply that seawater in leads is not a significant source of INPs, but that the biological growth occurring during this pre-melt onset period does not encompass microorganisms capable of high-temperature (>−15 °C) ice nucleation. All ice nucleation curves from all samples classified by date are shown in Fig. S4.† Furthermore, to estimate the abundance of airborne INPs or bioaerosols emitted from seawater, it is necessary to keep in mind that sea spray is only efficiently produced when the wind speeds exceed 4 ms^−1^ (ref. 105). Therefore, meteorological parameters must be considered in combination with marine biological activity proxies when interpreting ambient INP or bioaerosol data.

### 3.2 Physical, chemical and biological properties of potential natural Arctic aerosol sources

**Fig. 4:**
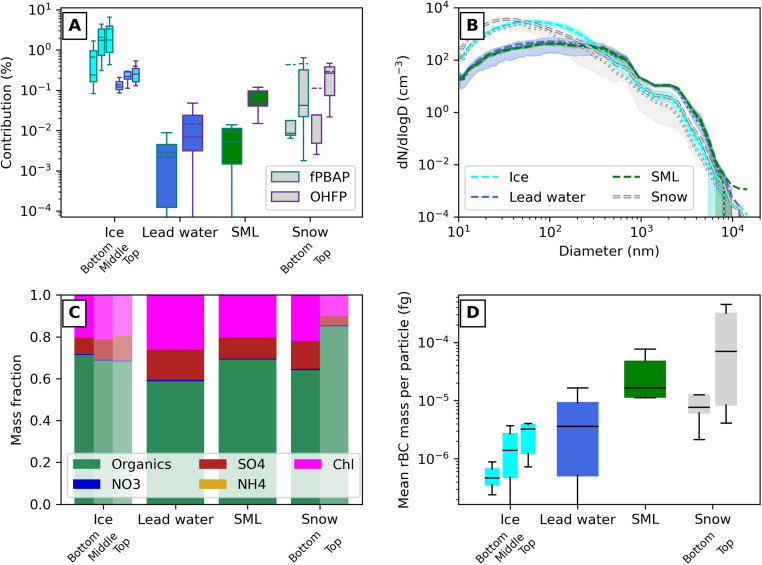
Overview of all analysed source samples sorted by source type. (A) Percentage contribution of fluorescent biological aerosol particles (fPBAPs) and other highly fluorescent aerosol particles (OHFPs) to the coarse-mode aerosol (particle diameter >0.8 μm) measured by the MBS. (B) Particle number size distribution measured by the SEMS and MBS. (C) Aerosol chemical mass fraction measured by the SP-AMS. (D) Refractory black carbon (rBC) mass concentration measured by the SP2, divided by the average total number concentration recorded for each sample. Aqua, blue, green, and grey indicate sea ice, lead water, SML, and snow source samples, respectively, in panels A, B and D. The results of the individual samples are shown in the ESI.†

The sea ice core samples showed the highest fPBAP contribution, followed by snow, SML, then lead water samples (see Fig. 4A). Samples taken from the upper section of the ice cores had a median contribution of fPBAPs to the coarse mode of 2%, several orders of magnitude higher than in other source types. For comparison, this is almost 100 times the contribution of fPBAPs found in ambient air at a mountain site on Svalbard during Arctic summer.^21^ The unexpected low contributions released from the SML can be explained by the sampling in the late spring season, during the start of the summer peak in productivity in the Arctic Ocean, which is known to be a period with low production of biological sea spray compared to later in summer.^106^ Wassmann and Reigstad^107^ also stated in 2011 that in a changing Arctic algae blooms may start earlier in the season, but their productivity may be lower due to disruptions in the ecosystem cycles. The OHFP contributions, probably being organic-coated sea salt particles,^59^ were around an order of magnitude higher for lead water, snow and SML samples compared to the corresponding fPBAP contribution. An exception is the sea ice core samples, where the OHFP contribution is almost an order of magnitude lower compared to the fPBAP contribution, indicating the high concentration of primary biological matter within the sea ice cores. Still, the OHFP contributions for the sea ice core samples are overall much higher than for the lead water samples. This can also be observed in the selected TEM images. Nearly all particles from the lead water and ice core samples shown in Fig. S5A and B,† respectively, contained NaCl, indicating that they are mainly composed of sea salt particles. Some particles also have organic coatings around the sea salt cores. Such organic coatings have a weaker contrast than sea salt cores and appear grey in the TEM images. Elemental mapping images (see Fig. S5C and D†) show that the particle cores consist of NaCl with minor amounts of Ca, Mg, K and S, all of which are components of seawater. In addition, C and O are distributed on the surface of the sea salt cores, indicating the presence of organic coatings. Note that the organic coatings only appear in the upper part of the particles in the TEM images because the lower part of the particles was shadowed by itself (shadowing effect). The sea salt particles with organic coatings were more abundant in the ice core sample (see Fig. S5B†) than in the lead sample (see Fig. S5A†).

Taking all samples together, a clear correlation was found between the contributions of OHFPs and fPBAPs (see Fig. S6A†), revealing that a higher emission of primary material coincides with a higher fraction of OHFPs or organic-coated particles. Here, snow was found to have the largest variability in OHFPs and fPBAPs among all samples, covering all the observed ranges, while sea ice cores, SML and lead water samples showed more cluster-like behaviour. This is an expected result, as snow is the most dynamic sample type in terms of sources. Not only could the sampled snow layer consist of a combination of precipitation from several different snow events with widely varying air mass origin, it could also have been mixed during storm events and may contain a contribution of particles from both local and remote sources. Interestingly, the OHFP spectra from SML showed a clear temporal transition, with a shift from C-dominating particles in the first half of the expedition, during the first ice station and transit, to a dominance of D-particles during the second half of the expedition (see Fig. S10A†) signalling a change in the organic composition of the SML, hence a change in their fluorescence spectra. This shift coincides with the onset of biological production in the ocean water sampled and the change from deeper to shallower waters, indicated by the increase in chlorophyll (see Fig. 3A). The sea ice core samples also showed distinct differences in the fluorescence spectra of both OHFPs and fPBAPs (see Fig. S10D and S9D,† respectively), with the first sampled sea ice core showing different spectral signals, indicating a different chemical and biological composition than the later sampled cores. This core was of similar length to several other cores, and we have no other variables that differentiate it from the others. It could be that this difference reflects the spatial variability of the sampling locations. An overview of fPBAP and OHFP spectra for all individual samples can be found in Fig. S9 and S10,† respectively. The snow and lead water samples, on the other hand, showed a large variability in OHFP spectra throughout the campaign (see Fig. S10B and C†). The mean size of fPBAPs within the lead water samples and snow was slightly larger and showed much more variability between the samples (values between 2 and over 5 μm in diameter, and 1 and 4 μm, respectively) compared to fPBAPs from sea ice cores where fPBAPs were only between 2 and 3 μm large (see Fig. S6B†). For the SML samples, the range of particle sizes was slightly smaller than for lead water (between about 2.5 to 4.5 μm in diameter), but still an overall larger and more variable population in size compared to the ice samples. Similarly, the morphology of the released fPBAPs was much more variable among all the other samples compared to the sea ice core samples (see Fig. S6C†), which could indicate that the variability in the atomised microorganisms was lower in the sea ice cores compared to other source samples.

**Fig. 5:**
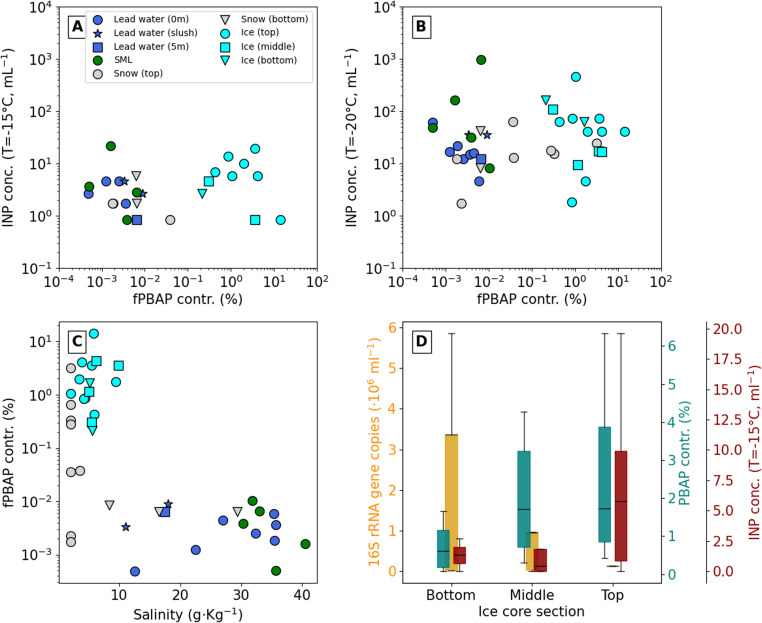
Relationship between bioaerosols, ice nucleating particles and salinity for all source samples and along the ice core thickness. (A and B) Ice nucleating particle (INP) concentration categorised by source type and section for two freezing temperatures, −15 °C and −20 °C, *versus* the contribution of fluorescent primary biological aerosol particles (fPBAPs) to the coarse-mode aerosol (particle diameter >0.8 μm). (C) fPBAP contribution to the coarse-mode aerosol as a function of sample salinity. (D) 16S rRNA gene copies per mL sample (prokaryote) and INP concentration at different sections of the ice core, and fPBAP contribution generated from these samples. Note that the cell and INP analysis was not done for all the ice core samples that were analysed for the fPBAP contribution.

The measured particle size distributions (see Fig. 4B) are mainly driven by the salinity of the sample, as previously observed,^108,109^ and the pressure setting of the atomiser. The particles released by the SML and lead water samples were generally larger compared to the snow and sea ice core samples (see Fig. S7†). Note that this result differs from the size patterns of the fPBAPs described above because since the fPBAPs do not consist of salt, their mean size does not depend on salinity. The source types with lower salinity (see Fig. S2 and Tables S2–S5†) required a higher pressure differential in the atomiser and therefore released a higher total number of particles, as demonstrated by the high peaks in the fine mode for these samples. The mean coarse-mode size also depends on salinity (see Fig. S7†). For most source types the size distribution is very robust, with low variability between samples. Snow samples exhibited the greatest variability in the shape of the size distribution, as well as in fPBAP and OHFP contributions. The range in salinity is narrow for snow and ice compared to other source types (see Fig. 5C), which means that the spread in size distribution is likely driven by non-sea-salt particles. Similarly, SML and lead water samples have the highest variability in salinity but very limited spread in size distribution. This could indicate that the characteristic distribution for each source sample type is highly influenced by, *e.g.*, the contribution of organics, in combination with the salinity.

Fig. 4C reveals that the sub-micrometer aerosol chemical composition of all sample types was dominated by organic matter, followed by chloride and sulphate (see Fig. S10† for the chemical composition of all individual samples). The top layer of snow showed a higher organic contribution compared to the underlying layer, while the three analysed layers of the sea ice core samples showed more or less the same contribution of organics. Interestingly, the relative amount of organics followed a similar pattern as the highly fluorescent particles (OHFPs, see Fig. 4A and S8†), which could be expected since especially large organic molecules are known to be fluorescent, and OHFPs are assumed to consist of salt particles coated with organics. The chloride mass fraction is consistently high for all source experiments, except in some of the top-layer snow samples (see Fig. S11†). Although sea salt does not flash vaporize efficiently at 600 °C, the significant chloride fraction observed in the source samples may partly be the result of the aerosolisation of saltwater solutions at sufficiently high concentrations to be detectable, but not measured quantitatively accurately.

The higher contribution of organics and OHFPs in the upper snow layers can be explained by the deposition of anthropomorphic aerosols, such as from incomplete combustion (for example, Freitas *et al.*^21^ also linked OHFPs to long-range transport and biomass burning). Particle concentrations may also be enhanced in the upper layers as a result of melting or compression of the snow layer in early summer. Fig. 4D confirms a higher contribution of black carbon mass in the upper snow layer compared to the underlying layer. This is in fact not surprising, as spring marks the end of the Arctic haze period.^7^ However, it is also possible that the signal we see is from contamination from deposited ship-stack or helicopter emissions. Although sampling locations were carefully chosen to always sample upwind of all engine emissions to minimise the risk of local contamination, it can not be fully excluded. A similar pattern as for snow can be observed, although at overall lower concentrations, for the different sea ice core layers. The upper layers show higher concentrations of black carbon, probably as a result of compressed snow. Interestingly, even the SML and seawater samples showed slightly elevated BC contributions, although these could be driven by few outliers and potential contamination (see Fig. S12A†).

The SP2 has previously been used to analyse snow^110,111^ and glacial ice core samples,^112,113^ but not for sea ice so far. Zanatta *et al.*^114^ showed that the presence of sea salt may alter the detection of rBC by the SP2, partly due to incandescence quenching by thick salt coatings on the rBC cores. The salinity of the ice and water samples in this study is higher than those in Zanatta *et al.*^114^ and probably affected the results. However, while Zanatta *et al.*^114^ observed a clear decrease in the concentration of rBC with increasing salinity, we see the lowest levels of rBC in ice, the source type with the second lowest salinity. Our generated particle concentrations were also several orders of magnitude lower, which probably dampened the effect. Furthermore, the rBC nebulisation efficiency may impact the observations.^115^ Together, these uncertainties make the absolute numbers of the detected rBC contributions less than reliable. However, given the orders of magnitude difference between sample types (Fig. 4D) and the discussion above, the result that the highest rBC contribution is found in snow samples, followed by SML, and that ice samples contain the lowest levels, is robust. Consequently, our samples indicate anthropogenic pollution in the high and not so pristine Arctic.

### 3.3 Biological properties and their link to ice nucleating particles

Although previous studies have shown a clear correlation between INPs and fPBAPs in the aerosol phase,^21,67,72^ this is not observed when comparing the aerosolised fPBAP contributions to INP concentrations measured in any of the source samples at freezing temperatures of −15 °C or −20 °C, respectively (see Fig. 5A and B). At *T* = −15 °C, fPBAP contributions are clearly higher in the sea ice core samples compared to the lead water and snow, with INP concentrations between 1 and 10 mL^−1^. These concentrations can be compared to those reported by Barry *et al.*,^95^ who observed INP concentrations of around 5000 mL^−1^ at −15 °C in seawater along the coastline of Utqiaġvik, Alaska, or by Irish *et al.*,^103^ who measured INP concentrations of about 100 mL^−1^ at −15 °C in seawater in the Canadian Arctic, both during the summer season. The INP concentrations we obtained are comparable to previous measurements of seawater in the North Atlantic in the early fall.^116^ This could indicate that the Arctic region in which we sampled was highly influenced by water inflow from the North Atlantic Ocean, or it simply reflects the season, and INP concentration would have been higher in the water in the end of summer. In Fig. 5A and B, some samples are not shown on the logarithmic scatter plot due to an INP concentration of 0. As expected, at the lower temperature of *T* = −20 °C, INP concentrations increase in magnitude and range from 1 to 10^3^ mL^−1^, but no relationship or correlation to the fPBAP contribution can be observed. The INP concentrations shown come from the samples in their liquid state, not from the aerosol they produce. Therefore, the lack of correlation could be due to selective aerosolisation, either caused by the applied aerosol generation method or by the particles themselves. At both freezing temperatures, the INP concentrations (and their spread) are similar across sources. When aerosolised, sea ice samples consistently had a much higher fPBAP contribution, often 100 to 1000 times more than lead water samples. It is known that sea-derived INPs around the Arctic often come from heat-labile sources.^79^ Despite similar concentrations of INPs, melted sea ice is likely a more efficient INP aerosol source than lead water. During the Arctic melt season, melt ponds cover a significant portion of the Arctic surface^117,118^ and thus may contribute significantly to the fPBAP and INP populations when aerosolisation mechanisms (*e.g.* wind) are present.

The aerosolisation process will depend on the salinity of the source sample since the salinity impacts the concentration and size of the salt particles produced.^109,119,120^ The interplay between film, jet and spume drops^17,121^ within the aerolisation will determine the size-dependent chemical^122–124^ and physical^125,126^ composition of the released salt particles, which in turn may impact how the biological and organic material will re-distribute among the produced particles. In this work, the mean coarse-mode size scales quasi-linearly with sample salinity (see Fig. S7†). However, for the contribution of fPBAPs, no correlation between the salinity of the samples was observed (see Fig. 5C). Even within the different sub-samples – sea ice, lead water or snow – the salinity did not impact the amount of released fPBAPs, confirming that the actual biological content of the sample and not the salinity is driving the amount of released fPBAPs. Interestingly, the snow samples showed the largest variation in fPBAPs, even at generally low salinity, reflecting the nature of snow that is produced by precipitation, with its content primarily influenced by air mass composition and scavenging of the aerosol as snow falls^70^ and subsequent microbial growth.^71^ These findings point towards blowing snow as a potential source for fPBAPs and INPs, especially since snow could easily be re-suspended and not depend on the formation of melt ponds first. However, this process will likely also depend on the season^24^ and the actual microphysical properties of the snow.

As mentioned above, the sea ice samples showed the highest fPBAP contributions. These contributions were not uniformly distributed along the thickness of the sea ice core, but rather showed a clear gradient with the fPBAP contributions increasing from the bottom to the top of the sea ice core (see Fig. 5D). The top of the sea ice core in the liquid phase also had significantly higher concentrations of INPs. Interestingly, the cell counts showed counter-intuitive behaviour compared to the INP and fPBAP values, with higher cell counts at the bottom of the sea ice core compared to the top. This is due to the migration and growth of microorganisms in brine channels that form at the interface of sea ice and seawater.^127^ These results indicate that the microbial communities in lead seawater have significantly different ice nucleating properties than those found in sea ice. Since we do see a covariation between cell counts in the liquid-phase lead water samples and fPBAP contribution to the aerosolised coarse mode (see Fig. 3), the opposite relation between cell counts and fPBAPs throughout the ice cores does not mean that we cannot detect the cells as fPABPs. However, it could corroborate the suggestion that microorganisms living on the sea ice surface are more likely to be aerosolised. The higher concentrations of fPBAPs at the top of the sea ice core reflect differences in the abundance of microorganisms found along the ice core.^128,129^ These findings point to possible evolutionary traits acquired by these organisms. In future work, differences in the composition of the microbial community^130^ will be further investigated using sequencing techniques.

## 4 Conclusions

During the ARTofMELT expedition, Arctic sea ice, snow, and seawater samples were collected at the start of the melt season to investigate their physical, chemical and biological properties.

Our study found notable differences in aerosol properties between these aerosol sources, with sea ice contributing the highest concentrations of fluorescent primary biological aerosol particles (fPBAPs) and other highly fluorescent particles (OHFPs). The observed increase in biological activity in seawater and the sea surface microlayer coincided with a rise in fPBAP and OHFP emissions; however, this increase in biological activity did not translate into an increase in ice nucleating particle (INP) concentrations within the water samples. The higher OHFP concentration was also linked to an increased organic mass fraction within the analysed particles. The enrichment of black carbon in the upper layers of the surface, particularly in snow, indicated a detectable anthropogenic influence even in the pristine Arctic environment. This could also be the reason for the increase in the organic mass fraction within the upper snow samples.

The counter-intuitive results regarding cell counts and aerosol emissions of melted sea ice samples highlight the importance of aerosolisation mechanisms that need to be taken into account when interpreting results of controlled or discrete sample analysis. Salinity did not appear to have a significant impact on fPBAP aerosolisation or ice nucleating activity. However, the enrichment of INPs and fPBAPs in sea ice samples, increasing toward the surface, points to melt ponds as a potential important source of INPs, although the exact aerosolisation process remains unclear.

This rich data set provides a valuable foundation for future research, offering the opportunity for detailed source-apportionment of natural aerosols and enhancing our understanding of aerosol–cloud interactions in the Arctic.

## Author contributions

The original concept of the study was developed by PZ. GF, JK, FM, LN, JC, CM, JS, and PZ were responsible for the acquisition of the main observational data. GF and JK performed the main data analysis and visualisation. GF, JK, FM, LN, CM, JS, KA, EA, and JC performed pre-processing and supporting data analysis. The manuscript text was written by PZ, GF, and JK, with input, contributions, and edits from all co-authors.

## Conflicts of interest

The authors declare that they have no conflict of interest.

## Supplementary Material

FD-258-D4FD00162A-s001

## Data Availability

The data of this study can be found at Kojoj *et al.* (2025)^131^ provided at the Data Centre of the Bolin Centre for Climate Research, Sweden.
