# Supplementary material for: A comprehensive characterisation of natural aerosol sources in the high Arctic during the onset of sea ice melt
Source: Faraday Discuss. 2024 Nov 25;258:120–46. doi: 10.1039/d4fd00162a (PMC11877279; doi:10.1039/d4fd00162a)
Supplement: FD-258-D4FD00162A-s001 [file FD-258-D4FD00162A-s001.pdf]

Supplementary Information (SI) for Faraday Discussions.
This journal is © The Royal Society of Chemistry 2025

Supplementary information for "A comprehensive characterisation of natural aerosol sources in the high Arctic during the onset of sea ice melt"

Gabriel Pereira Freitas[1,2,*], Julia Kojoj[1,2,*], Camille Mavis[3], Jessie Creamean[3], Fredrik Mattsson[1,2], Lovisa Nilsson[4], Jennie Spicker Schmidt[5], Kouji Adachi[6], Tina Santl-Temkiv[5], Erik Ahlberg[4], Claudia Mohr[1,2,7,8], Ilona Riipinen[1,2], and Paul Zieger[1,2,*]

[1]Department of Environmental Science, Stockholm University, Stockholm, Sweden
[2]Bolin Centre for Climate Research, Stockholm University, Stockholm, Sweden
[3]Department of Atmospheric Science, Colorado State University, USA
[4]Department of Physics, Lund University, Sweden
[5]Department of Biology, Aarhus University, Denmark
[6]Department of Atmosphere, Ocean, and Earth System Modeling Research, Meteorological Research Institute, Tsukuba, Japan
[7]PSI Center for Energy and Environmental Sciences, Paul Scherrer Institute, Villigen, Switzerland
[8]Department of Environmental Systems Science, ETH Zurich, Zürich, Switzerland
[*]Contributed equally to this work.

Correspondence: Paul Zieger (paul.zieger@aces.su.se)

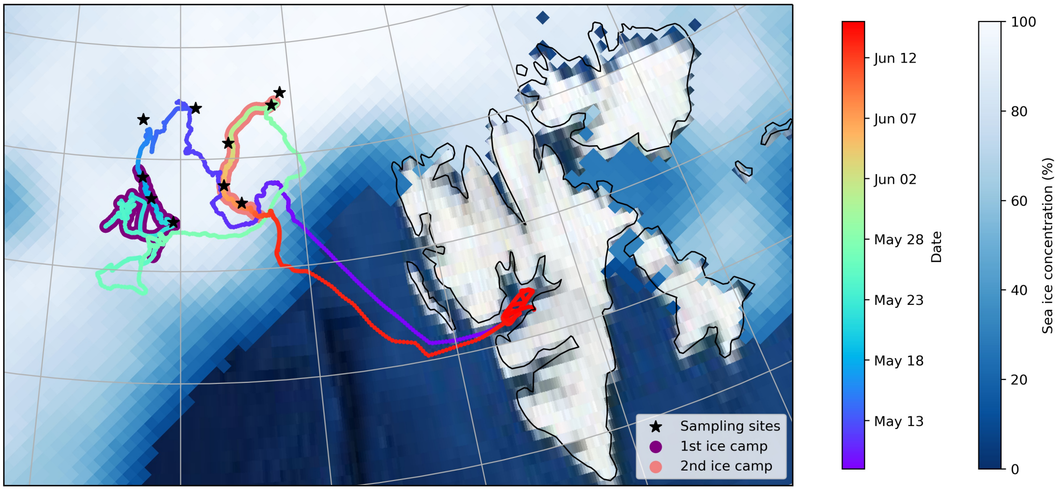

Figure S1. ARTofMELT cruise track and sampling sites. The sea ice concentration are shown for the 12th of June 2023 (satellite data from the University of Bremen, https://seaice.uni-bremen.de/sea-ice-concentration/amsre-amsr2/, last accessed 21 November 2024). Start and end of the expedition was Longyearbyen, Svalbard. The stars indicate sampling sites that were either on the main ice station floes or the remote ice stations accessed by helicopter

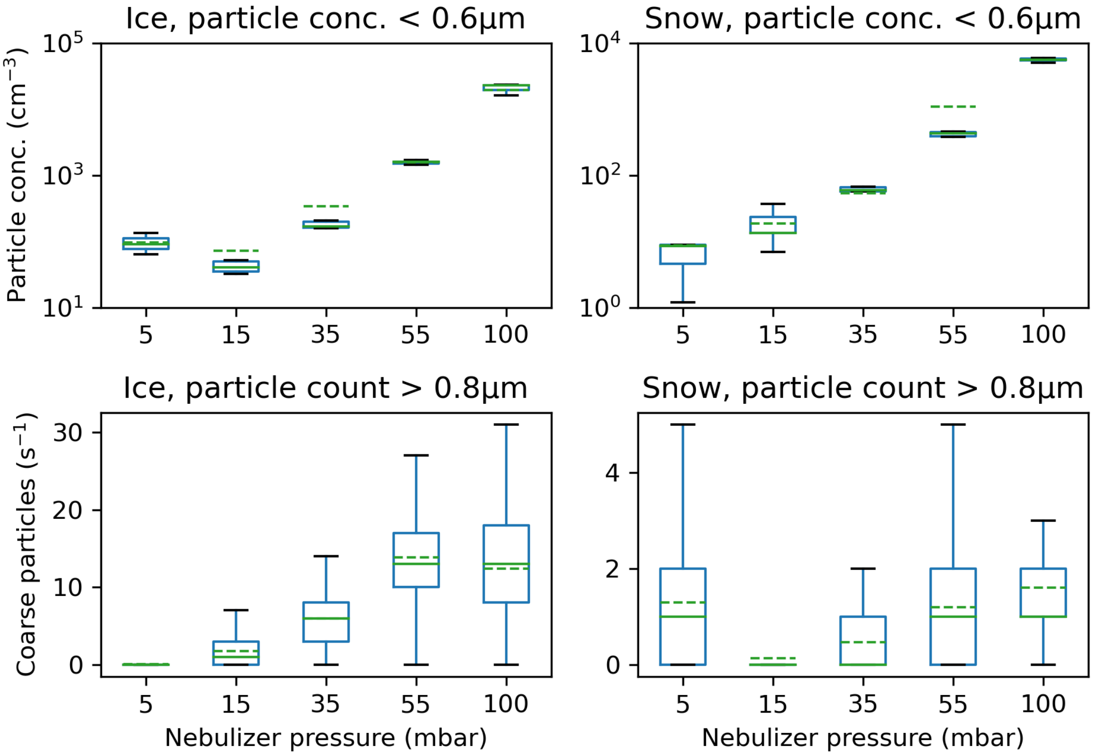

Figure S2. Number of particles generated as a function of the nozzle pressure set in the aerosol generator for the ice and snow samples. Shown are the particle concentrations versus the nozzle pressure which determines the air flow rate. The upper panels show the particle concentrations for sub-micron particles below 0.6 μm (A and B, measured by the SEMS), while the lower panels show the coarse-mode particles concentrations above 0.8 μm (C and D, measured by the MBS). Note the different y-axis scales between the different panels.

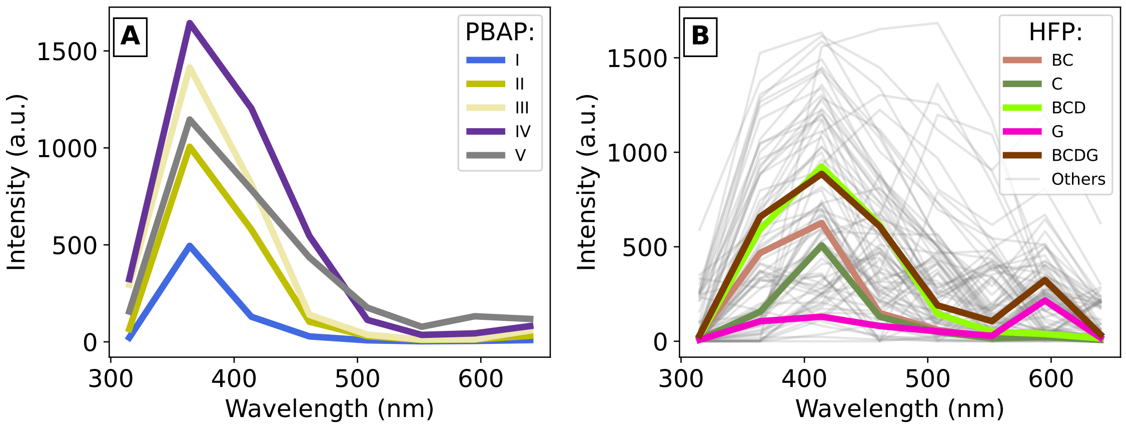

Figure S3. Average fluorescence emission spectra for all measured fPBAP and OHFP spectra. A) Average spectra of fPBAP particles (mean over the entire dataset) for the five fPBAP classes. B) Average (mean) fluorescence emission spectra for the five most abundant OHFP particles (coloured lines) and all the other remaining OHFP particles (39.6% of all analysed particles; grey lines).

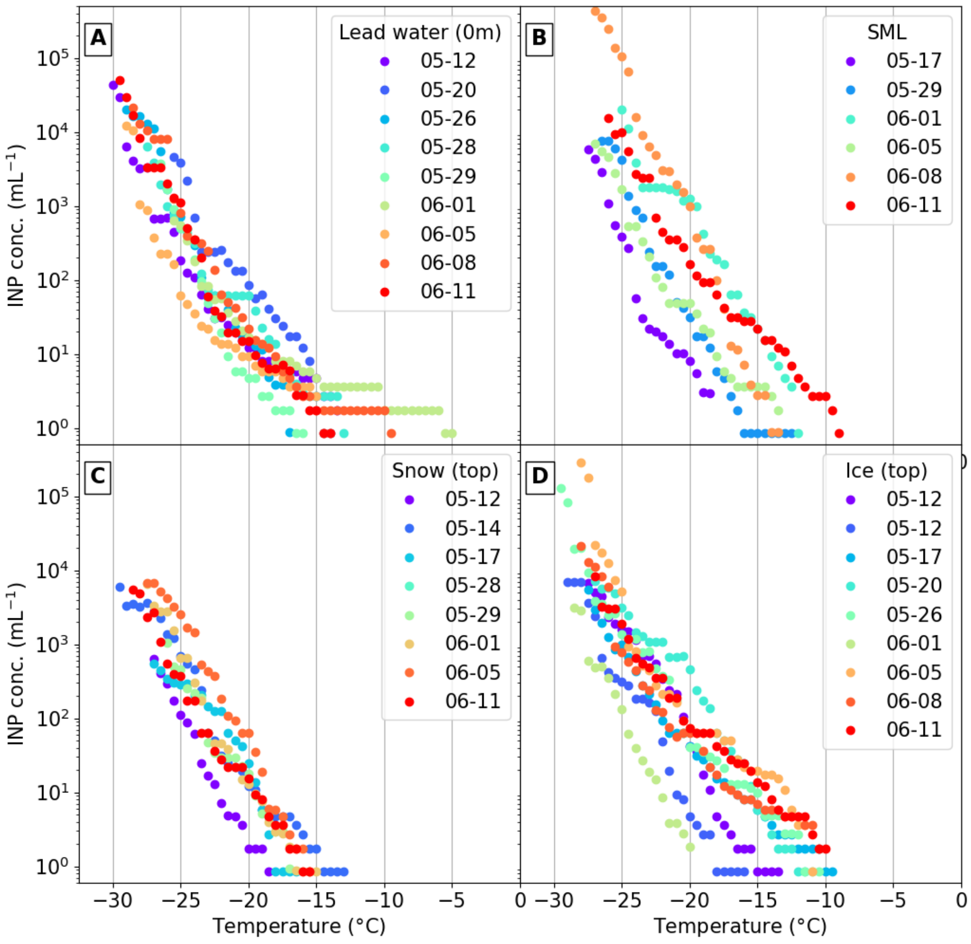

Figure S4. Ice nucleating particle concentration versus temperature. A) Surface seawater, B) sea surface microlayer (SML), C) top snow layer and D) top ice layer. The colour-code states the date of sample taking.

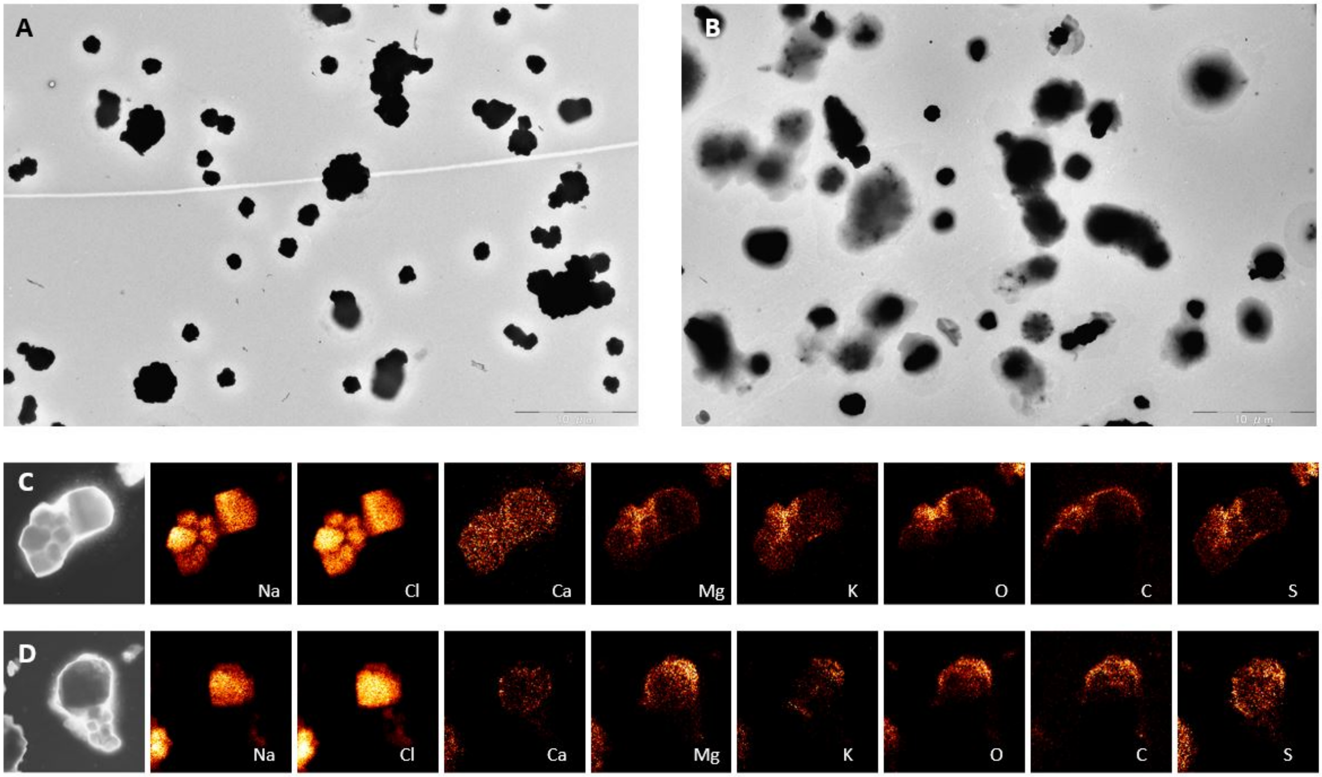

Figure S5. Example from the transmission electron microscopy (TEM) analysis and the elemental mapping using an energy-dispersive X-ray spectrometer. A) Particles atomised from lead water soure samples. B) Particles atomised from the top portion of an sea ice core sample. Scale bars indicate 10 μm. Panels (C) and (D) show the elemental mapping for two particles of the lead water sample.

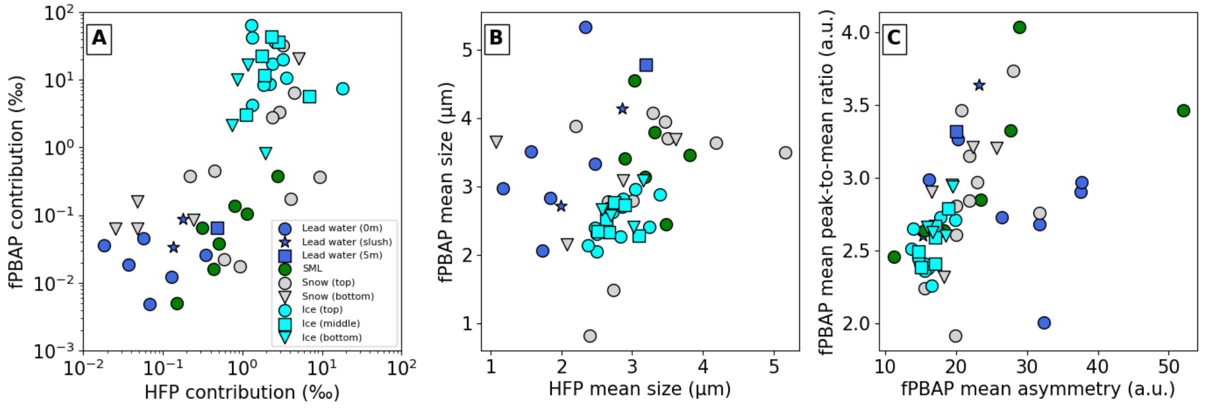

Figure S6. Key parameters from the multiparameter bioaerosol sensor (MBS) for the different source samples. A) Fluorescent primary biological aerosol particle (fPBAP) vs. the highly fluorescent particles (OHFP) contributions to coarse mode aerosol (particle diameter > 0.8 μm). B) Mean size of fPBAP and OHFP particles. C) Mean shape parameters of fPBAP: peak-to-mean ratio vs. asymmetry.

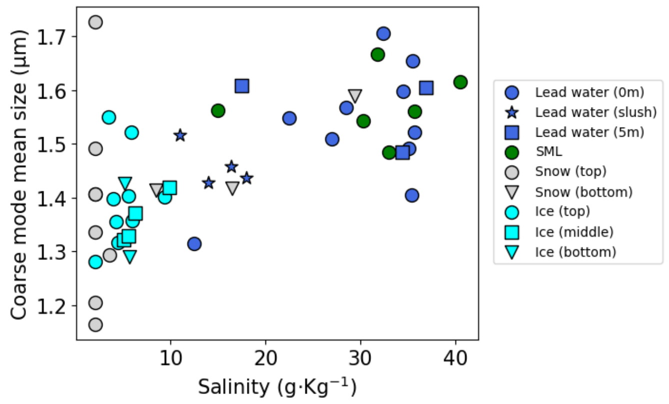

Figure S7. Comparison between coarse mode mean size to salinity. A comparison, for every source sample type, between the coarse mode aerosol (optical diameter > 8 μm) and the salinity (g·kg^{-1}) of the sample.

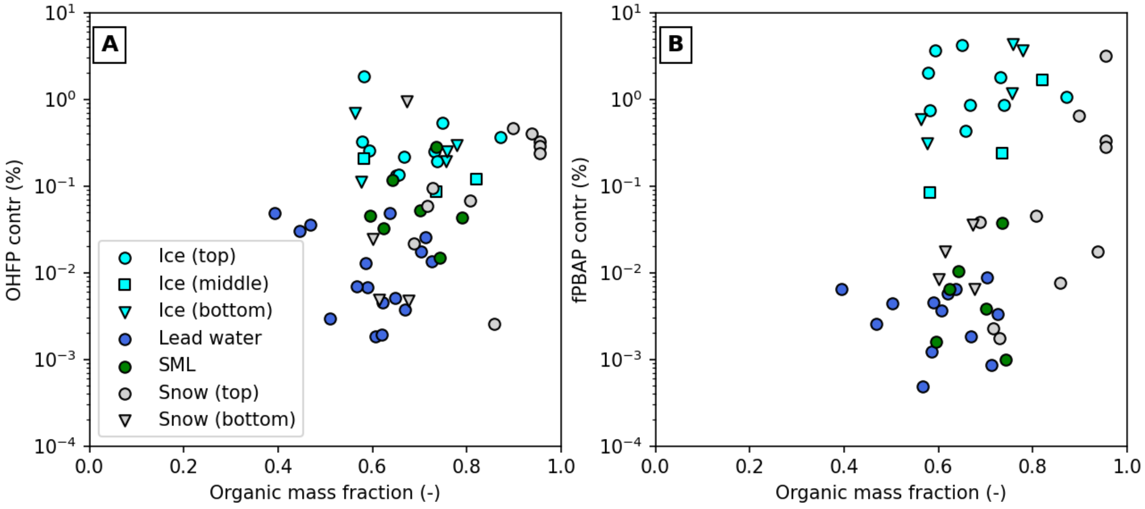

Figure S8. Contibution of fluoresent particle types versus organic fraction in all aerosolised samples. Percentage contribution of A) fluorescent biological aerosol particles (fPBAP) and B) highly fluorescent aerosol particles (OHFP) to coarse-mode aerosol, obtained from the MBS, plotted versus the average fraction of organics in each measured by the SP-AMS.

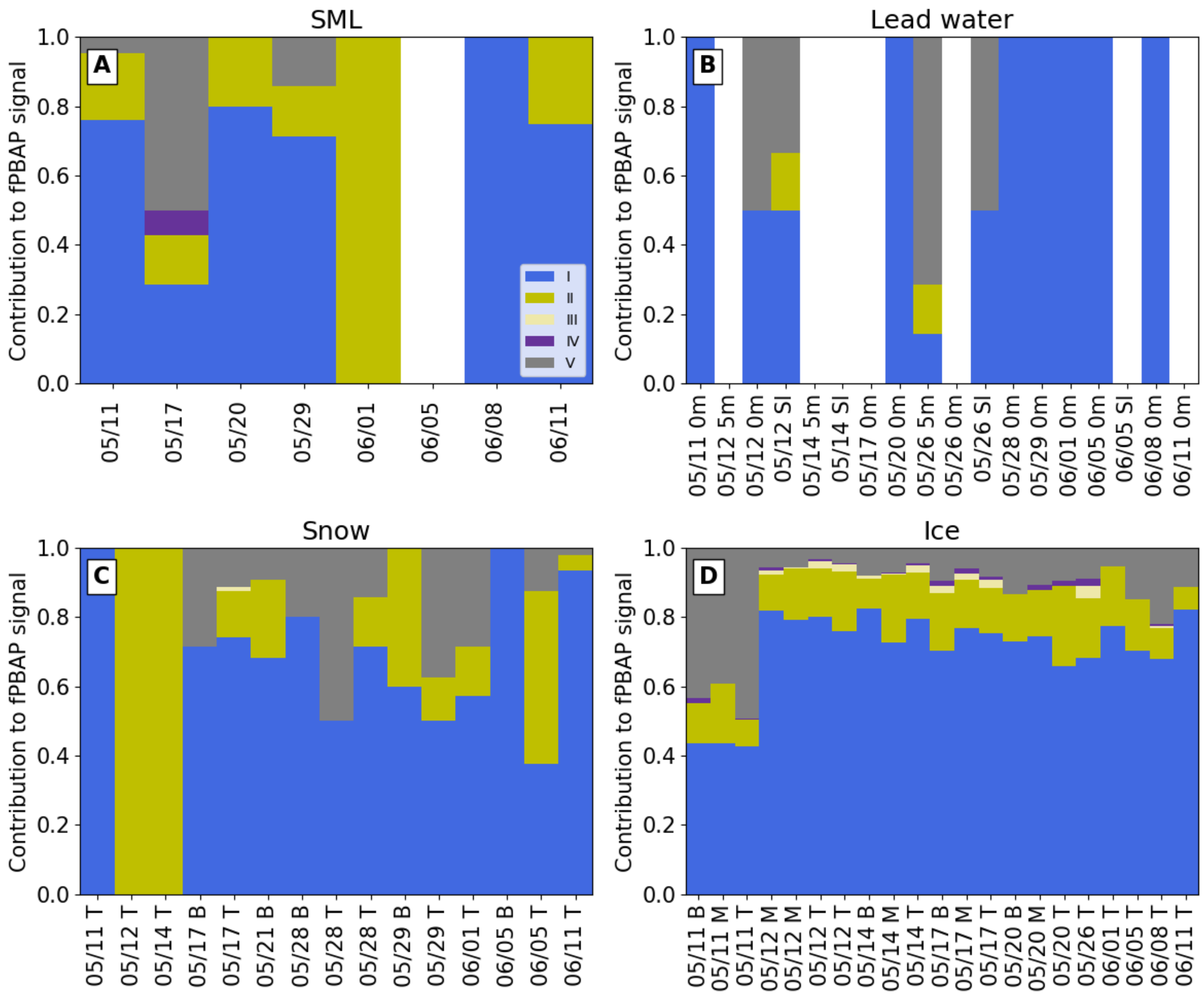

Figure S9. Types of fluorescent primary biological aerosol particles (fPBAP) for the different source types identified with the MBS. More details on the sampling dept, layer and the corresponding chlorophyll-a and salinity values are given in the Tables S2 to S5.

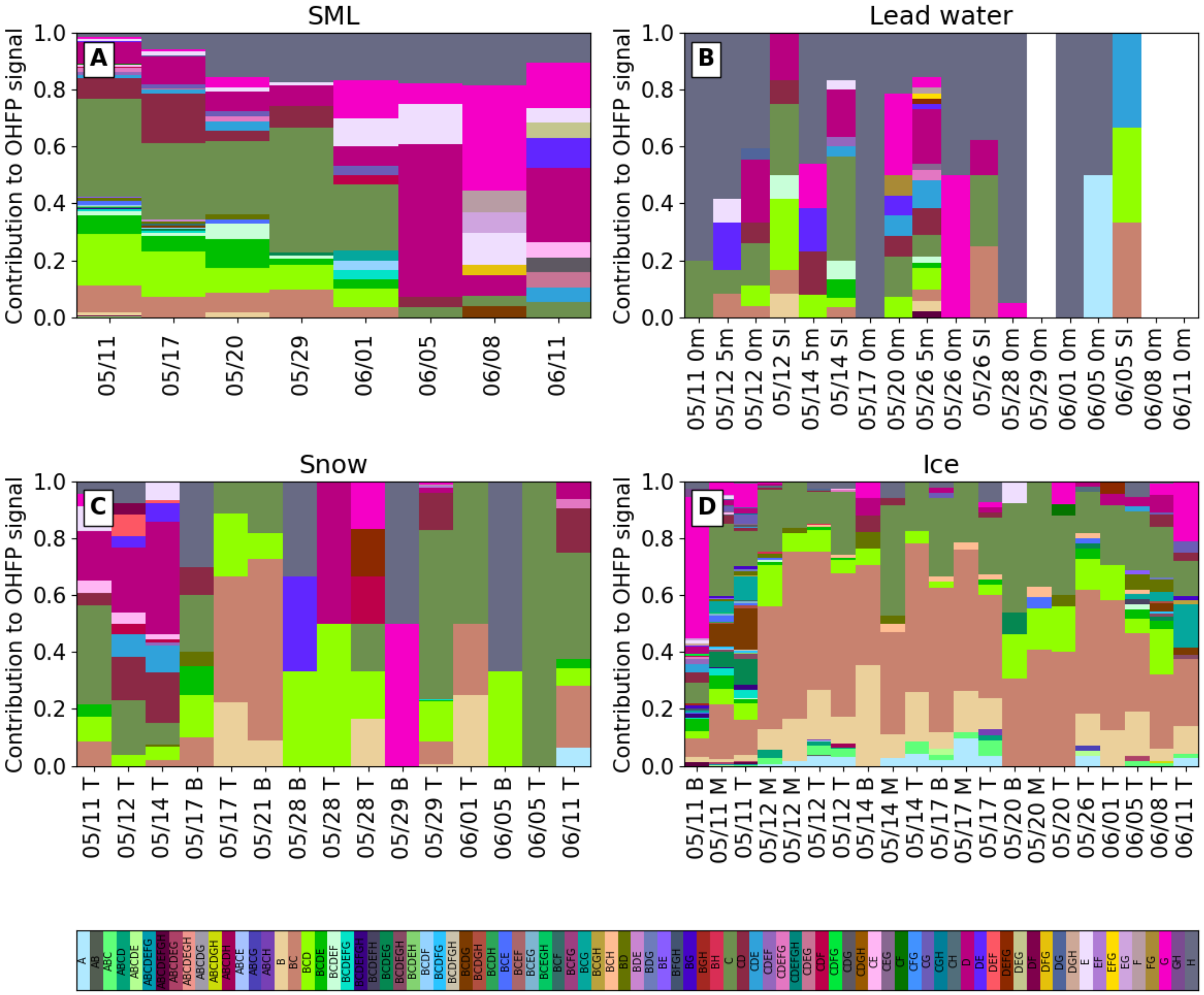

Figure S10. Types of highly fluorescent particles (OHFP) for the different source types identified with the MBS. More details on the sampling dept, layer and the corresponding chlorophyll-a and salinity values are given in the Tables S2 to S5.

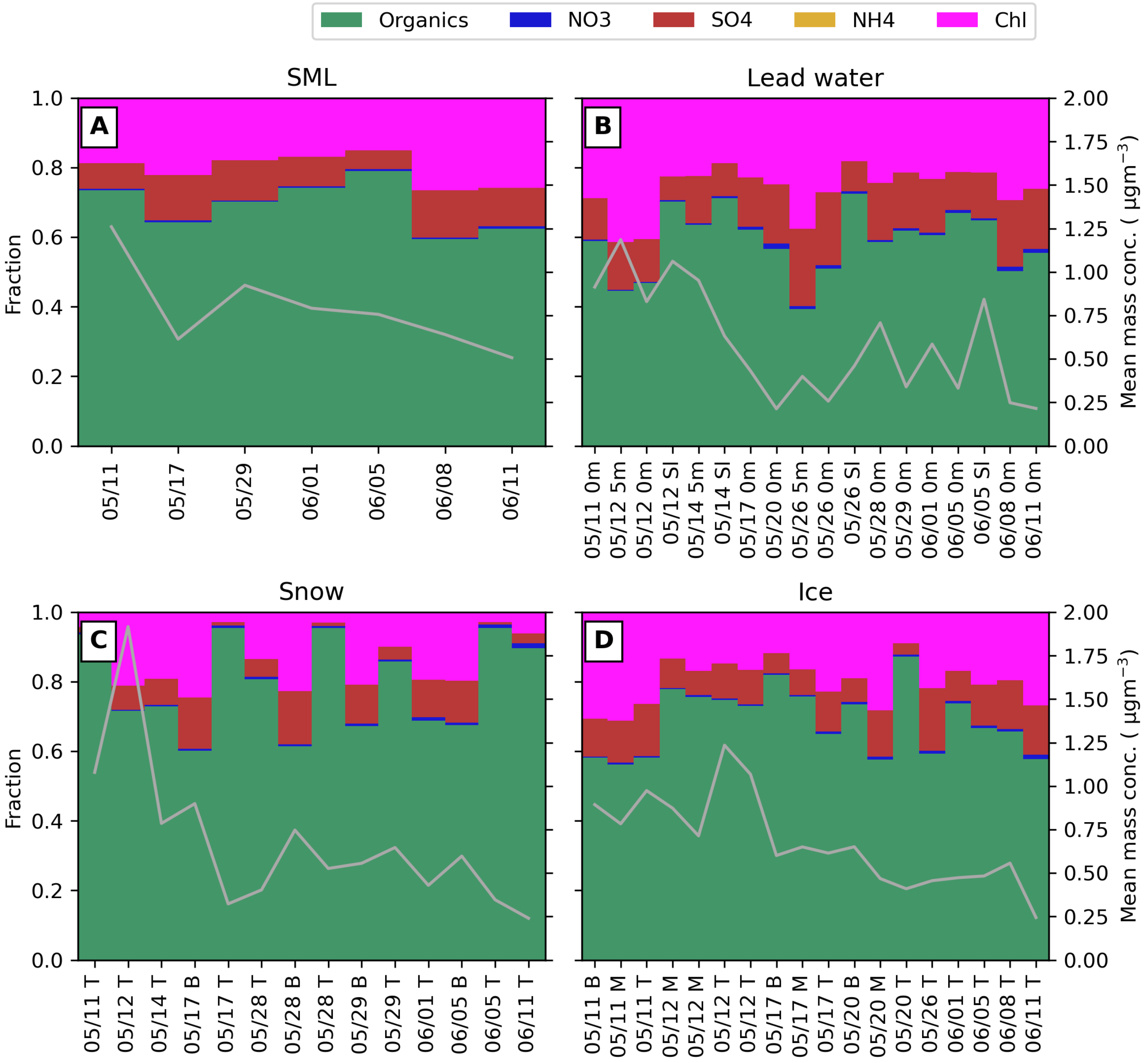

Figure S11. Chemical mass fraction for the different source samples measured by the SP-AMS. Mass fractions (left axis) are representative for sub-micron non-refractory aerosol. The mean measured mass concentration is shown on the right axis. More details on the sampling dept, layer and the corresponding chlorophyll-a and salinity values are given in the Tables S2 to S5.

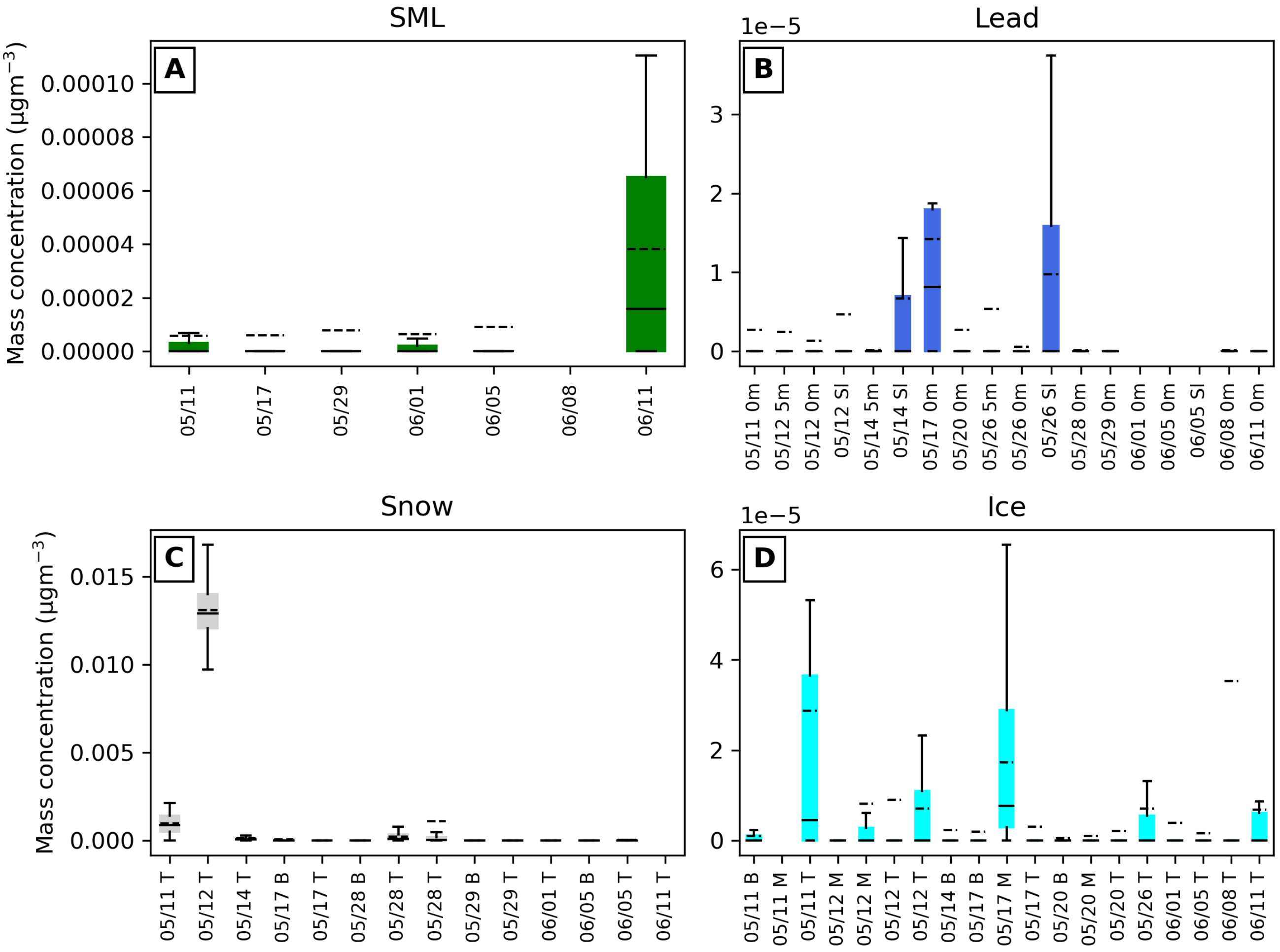

Figure S12. Refractory black carbon mass concentration for the different source samples measured by the SP2. More details on the sampling dept, layer and the corresponding chlorophyll-a and salinity values are given in the Tables S2 to S5.

Table S1. Number of samples collected for each source sample type (Total samples), and the respective number of samples analysed by specific methods. One analysed brine sample is not included in this table.

	Source type			
	SML	Leadwater	Snow	Ice
Total samples	8	18	15	21
MBS	8	18	15	21
SP2	8	18	15	21
SMPS	8	18	15	21
SP-AMS	8	18	15	21
TEM	8	11	10	16
16s / 18s	6	11	0	4
INP	6	18	11	15
Physiochemical	6	17	10	14

Table S2. List of all snow samples together with corresponding sampling layer, sampling date and the measured salinity. The salinity could not be determined from all samples.

Sample number	Sampling section	Sampling date (month-day)	Salinity (gkg^{-1})
1	Top	05-11	
2	Top	05-12	<2.0
3	Top	05-14	<2.0
4	Top	05-17	<2.0
4	Bottom	05-17	8.4
5	Bottom	05-21	
6	Top	05-28	
6	Top	05-28	
7	Top	05-29	<2.0
7	Bottom	05-29	29.4
8	Top	06-01	3.5
9	Bottom	06-05	16.5
9	Top	06-05	<2.0
10	Top	06-11	<2.0
11	Top	05-28	<2.0

Table S3. List of all sea ice core samples together with corresponding sampling layer, sampling date and the measured salinity and chlorophyll-a concentration. The last column gives the length of the analyzed core sample. The salinity and chlorophyll-values could not be determined from all samples..

Sample number	Sampling layer	Sampling date (month-day)	Salinity (gkg^{-1})	Chlorophyll-a (μgL^{-1})	Core depth (cm)
1	Top	05-11			0-10
1	Middle	05-11			50-60
1	Bottom	05-11			141-151
2	Top	05-12	5.8	0.03	0-20
2	Middle	05-12	9.9	0.44	20-35
3	Middle	05-12	5.0	0.44	20-35
3	Top	05-12	9.4	0.03	0-20
4	Middle	05-14			20-40
4	Top	05-14			0-20
4	Bottom	05-14			40-64
5	Bottom	05-17	5.1	0.02	119-139
5	Middle	05-17	6.2	0.03	60-80
5	Top	05-17	3.9		0-19
6	Bottom	05-20	5.6	0.55	143-163
6	Top	05-20	<2	0.04	0-20
6	Middle	05-20	5.5	0.03	70-90
7	Top	05-26	5.5	0.06	0-20
8	Top	06-01	4.2	0.13	0-20
9	Top	06-05	4.4	0.08	0-20
10	Top	06-08	5.9	0.07	0-20
11	Top	06-11	3.4	0.21	0-20

Table S4. List of lead water samples together with corresponding sampling layer, sampling date and the measured salinity and chlorophyll-a concentration. The salinity and chlorophyll-values could not be determined from all samples.

Sample number	Sampling layer	Sampling date (month-day)	Salinity (gkg^{-1})	Chlorophyll-a (μgL^{-1})
1	0 m	05-11		
2	Slush	05-12	18.0	
2	0 m	05-12	32.4	0.04
2	5 m	05-12	36.9	0.25
3	Slush	05-14	16.4	
3	5 m	05-14	34.4	0.13
4	0 m	05-17	35.1	0.07
5	0 m	05-20	12.5	0.05
6	5 m	05-26	17.5	0.06
6	Slush	05-26	11.0	0.34
6	0 m	05-26	34.5	0.06
7	0 m	05-28	22.5	0.3
8	0 m	05-29	35.4	0.85
9	0 m	06-01	35.7	1.63
10	Slush	06-05	14.0	2.84
10	0 m	06-05	35.5	1.92
11	0 m	06-08	27	1.66
12	0 m	06-11	28.5	2.57

Table S5. List of all sea surface microlayer samples together with corresponding sampling layer, sampling date and the measured salinity. The salinity was not measured for all samples.

Sample number	Sampling date (month-day)	Salinity (gkg^{-1})
1	05-11	
2	05-17	39.5
3	05-20	
4	05-29	30.3
5	06-01	35.7
6	06-05	15.0
7	06-08	40.5
8	06-11	33

Table S6. Size statistics of fPBAP and OHFP per source type. These account for all samples of each source type combined.

	fPBAP Mean Size(μm)	fPBAP STD Size (μm)	fPBAP Total Number	OHFP Mean Size (μm)	OHFP STD Size (μm)	OHFP Total Number	Coarse Mode Total Number
SML	3.35	1.37	100	3.15	1.21	880	946398
Snow	3.05	1.15	241	2.91	1.41	462	511312
Lead	4.05	2.32	32	2.57	1.52	204	1438011
Ice	2.21	0.73	26741	2.64	0.95	3747	890968
